# Drying Kinetics and Desorption Isotherms of *Chondracanthus chamissoi* (C. Agardh) Kützing, 1843 at Different Temperatures

**DOI:** 10.1155/ijfo/6647658

**Published:** 2026-07-01

**Authors:** Alessandra Palomino-Cáceres, Paul M. Baltazar-Guerrero, Oscar Reategui

**Affiliations:** ^1^ Marine Biology Program, Faculty of Environmental Sciences, Scientific University of the South, Lima, Peru; ^2^ Sustainable Aquaculture Research Group (GIAS); Marine Crops Research Laboratory (LICMA), Vice-Rectorate for Research, Development and Innovation, Scientific University of the South, Lima, Peru; ^3^ Research Group on Characterization, Transformation and Sustainability of Natural Resources in Peru (CTS Group), Vice-Rectorate for Research, Old Pan-American Highway South Km 19. VES. Lima, Scientific University of the South, Lima, Peru

**Keywords:** carposporophyte, drying process, Peru seaweed, red macroalgae, tetrasporophyte

## Abstract

This study is aimed at modeling the water sorption isotherms and air‐drying kinetics of *Chondracanthus chamissoi* in tetrasporophyte and carposporophyte stages. A convective dryer was used for the desorption experiments at 25°C, 40°C, and 55°C, using the GAB (Guggenheim–Anderson–de Boer), BET (Brunauer–Emmett–Teller), Halsey, and Oswin equations. The best fit for both phases was achieved using the GAB model, as evidenced by the highest *R* ^2^ values (0.9988 for the tetrasporophyte at 55°C and 0.9985 for the carposporophyte at 40°C) and the lowest RMSE values (0.0058 for the tetrasporophyte at 55°C and 0.0075 for the carposporophyte at 40°C). Drying experiments were conducted in a climatic chamber at four different air temperatures: 40°C, 50°C, 60°C, and 70°C. The drying behavior was analyzed using Newton, Henderson–Pabis, Page, and Modified Page models. When comparing experimental moisture ratio values with calculated MR values, the Modified Page model was found to have the best fit with the same statistical test for both phases. Therefore, both equations efficiently simulate the *C. chamissoi* drying process and are an excellent tool for estimating drying time.

## 1. Introduction

*Chondracanthus chamissoi* (C. Agardh) Kützing, commonly known as “yuyo” or “chicorea de mar” by local fishermen [1], is found along the South Pacific coast from Perú (5°S) to Chile (42°S) [2]. This red seaweed is purchased because of its high protein content, significant vitamins, microelements, and antioxidant activity [3, 4]. It is also known as a great raw phycocolloid source for thickeners, emulsifiers, and gelling agents [5, 6] and as a potential functional food for mucosal barrier function in mammals [7].

The life cycle of *C. chamissoi* has three phases, in which the tetrasporophyte releases haploid spores, or tetraspores, that later settle and grow into a female or male gametophyte. When the male fertilizes the female gametophyte, the female turns into a carposporophyte with cystocarps on the pinnules, which release diploid carpospores that later turn into a tetrasporophyte seaweed [8].

In 2019, 1511 tons of *C. chamissoi* was collected in Peru [9], and most of the product was exported to China [10]. It is of great interest to continue to develop the culture of this seaweed in Peru due to its presence and growing market demand [1, 11]. Therefore, it is important to study processing methods to obtain the best quality product for consumers.

One fundamental step during processing is drying [12]. The drying process can comprise the greatest usage of energy among the postharvesting steps, by removing the water content of the product to reduce microorganism growth and deteriorating chemical reactions [13]. Drying also contributes to minimizing the size and volume of the product to increase transport and storage efficiency [14].

Traditionally, dehydration has been performed under direct sunlight, also called natural convection, in which case the process relies on environmental conditions [15]. More recently, various drying techniques have become available, such as convective drying, spray drying, freeze drying, and osmotic dehydration, among others [16]. One of the simplest, more commonly used and low‐cost methods in the industry is convective drying with hot air [17], which is not only a simple operation but also allows user control of important drying parameters such as temperature, relative humidity, air velocity, and time [14].

Drying kinetics studies allow the user to manipulate these dimensions and determine the effect they may have on the drying process, especially on drying time [18]. Mathematical equations are used to describe water loss and to simulate the drying process with the established parameters of the model to gather more accurate and specific information on the product of interest [19]. These equations result in mass transfer as a function of a given condition such as temperature, humidity, density, and pretreatments, among others [20].

The equilibrium moisture content (EMC) is the value of moisture, commonly expressed on a dry basis, that a product achieves after exposure to a given temperature and relative humidity, meaning the time at which the product does not gain or lose any more water [21]. This occurs when the pressure of the water in the air and on the product’s surface is the same, and thus, no more molecules are exchanged. It does not, however, mean that both the air and the algae have the same water content [22].

The method most utilized to determine the EMC was standardized by Cost‐Project 90, performed by Follet et al. [23], in which 32 laboratories worked in partnership to find the best way to obtain isotherm measurements for different food products. It is important to calculate this value to predict the sorption behavior and to define the final moisture content of the process to achieve the most adequate storage conditions to obtain the best product quality. It can also bring stability to the algae and prevent early deterioration [24].

Regarding the drying kinetics, the mathematical equations used are aimed at predicting the drying time. This information is valuable because it gives the user a set time and prevents overdrying and overusage of power [25]. The semiempirical Newton, Modified Page, and Henderson–Pabis models are currently used because they provide a representation of the mass transfer [26].

Similar research on drying processes has been conducted for other red seaweeds such as *Gracilaria chilensis* [15, 18, 25, 27], *Gelidium sesquipedale* [19], and *Euchema cottoni* [28], as well as brown seaweeds, including *Bifurcaria bifurcata* [29], *Fucus vesiculosus* [30], *Himanthalia elongata* [31], and *Macrocystis pyrifera* [17], and green macroalgae such as *Ulva ohnoi* [24], providing end of drying time, the EMC, and avoiding the inefficient use of energy [27]. Considering the growing demand and lack of information on *C. chamissoi*, the present study was carried out.

Therefore, this study is aimed at determining the desorption isotherms at 25°C, 40°C, and 55°C and drying kinetics at 40°C, 50°C, 60°C, and 70°C of the rhodophyte *C. chamissoi* for carposporophyte and tetrasporophyte stages and at finding the best mathematical model for both phenomena.

## 2. Methodology

The nomenclature and units related to the drying kinetics and mathematical modeling are summarized in Table 1. These parameters are organized into three primary groups: moisture state variables, isotherm modeling parameters, and statistical and operational metrics.

**Table 1:** Nomenclature used for drying experiments.

Symbol	Meaning
*X*_*e*_	Equilibrium moisture content; g water/g dry matter (d.m.)
*X*_*t*_	Moisture content at any time; g water/g d.m.
*X*_0_	Initial moisture content; g water/g d.m.
*D*	Dry mass; g
*M*_*e*_	Equilibrium moisture content experimental mass of algae; g
*M*_*o*_	Monolayer value
*a*_*w*_	Water activity
*C*	Parameter of isotherm model
*K*	Parameter of isotherm model
MR	Moisture ratio
*r* ^2^	Coefficient of determination
RMSE	Root mean square error
SSE	Sum square error
*t*	Drying time; min
*T*	Temperature; Kelvin

### 2.1. Raw Material

Fresh *C. chamissoi* seaweed (initial moisture content of 9.76 g water/g d.m.) was purchased from the Cooperativa de Trabajadores Pesqueros Artesanales Algas Marinas (COTRAPALMAR) and processed at the laboratory of Algaex SA company, both located in the San Andrés district, in the Pisco, region of Ica, Peru. The samples were thoroughly washed with fresh water to remove epiphytes and sand and were separated by reproductive stage into a carposporophyte group and a tetrasporophyte group. The samples were stored at −20°C for up to 1 week prior to subsequent treatment.

### 2.2. Sorption Isotherm Experiments

The EMC of the algae was determined using the static gravimetric methodology by European Project COST 90 [23]. For this method, small samples of algae (0.5 ± 0.02 g) in sealed jars with a specific water activity (*a*_*w*_) were used. One quarter of each glass jar was filled with a saturated salt solution, giving a fixed humidity [19]. The saturated salt solutions employed were CH_3_COOK, MgCl_2_, KI, NaCl, and KCl, which were prepared according to the recommendation of Greenspan [32] at 25°C, 40°C, and 55°C, to obtain a range of 0.1755–0.8434 *a*_*w*_. In order to inhibit microbial growth, a small quantity of thymol was also placed in the glass flasks in which water activity was greater than 0.5 [25, 29].

The samples were weighed at regular intervals until a constant weight (±0.0005 g) was achieved after three readings in a row, with an analytical balance (Pioneer TM Analytical PX224). Dry mass samples were determined after drying in a hot air oven at 105°C until a constant weight was achieved [19, 31]. All experiments were carried out in triplicate.

The equation to calculate the EMC (% dry basis) was as follows [24]:

(1) Me=Me,g−DD,

where *M*_*e*,*g*_ is the equilibrium mass of algae in grams and *D* is the dry weight of the sample in grams.

### 2.3. Modeling of Desorption Isotherms

Three models were calculated to fit the experimental EMC at different water activities: GAB (Guggenheim–Anderson–de Boer) (Equation 1) and BET (Brunauer–Emmett–Teller) (Equation 2) for *a*_*w*_ < 0.5, modified BET (Equation 3), Hasley (Equation 4), and Oswin models [19, 24, 29].

(2) Xe=XmoCKaw 1−Kaw1+C−1aw,

(3) Xe=XmoCaw 1−aw1+C−1aw,

(4) Xe=Aln1/aw1/B,

(5) Xe=Aaw1−awB.

Model fitting was performed by the parameter values to minimize the sum of square errors between the modeled and the experimental data. These mathematical calculations were carried out using the solver function of Microsoft Excel with the GRG nonlinear solver method [24, 30, 33]:

(6) Obj=∑m=1n∑Xexp−Xcal2,

where *m* is a given dataset and the Obj function is minimized by the variation of the model constants.

### 2.4. Drying Experiment

Drying experiments were carried out in a climatic chamber (HPP110, Memmert, Germany) at four air temperatures: 40°C, 50°C, 60°C, and 70°C [25]. Relative humidity 35*%* ± 0.5*%* and air velocity remained constant throughout all the experiments. Seaweed samples of 10 g ± 0.03 and similar sizes were selected and arranged in a lipped cylindrical glass container and weighed using a digital electronic balance (Pioneer TM Analytical PX224) every 10 min for 80 min and then every 20 min. The experiments were finished at the point of constant weight, or equilibrium condition [25].

Dry mass was attained once again in the hot air oven at 105°C until constant weight [19, 31].

### 2.5. Drying Kinetics Model

The experimental data were plotted for the four temperatures as a dimensionless variable moisture ratio (MR) versus time (minute):

(7) MR=Xt−XeX0−Xe,

where *X*_*t*_ is the moisture content at any time *t*, *X*_*e*_ is the EMC, and *X*_0_ is the initial moisture content, and all are expressed as g water/g dry matter. The experimental data were fitted to three different models (Table 2).

**Table 2:** Models used for drying experiments.

Model	Equation
Newton	MR = exp(−*k*_1_ *t*)
Modified Page	$\text{MR}=\exp\left(-{\left(k_{2}t\right)}^{n}\right)$
Henderson–Pabis	MR = *α*exp(−*k*_3_ *t*)

*Note:* *k* (min^−1^) and *n* (−) are model parameters, and *t* is drying time (min).

The kinetic (*k*_1_, *k*_2_, and *k*_3_) and empirical (*n*, *a*) parameters of each model were also identified using the solver equation [24].

### 2.6. Statistical Analysis

All experiments were carried out in triplicate. The goodness of fit of the mathematical models was evaluated using the coefficient of determination (*R* ^2^) and the sum of squared errors (SSEs) (Equation 8) and root mean square error (RMSE) (Equation 9).

(8) SSE=1N∑i=1NXexp−Xcal2,

(9) RMSE=1N∑i=1NXexp−Xcal212/,

where *N* is the number of samples, *X*_exp_ is the experimental data, and *X*_*c* *a* *l*_ is the calculated data values for both the isothermal and drying kinetics models. To evaluate the temperature effect, the ANOVA test was carried out for the average of the empirical and kinetic constants of the models, followed by a multiple range test (MRT) considering significant values *p* ≤ 0.05 (Statgraphics 19X64).

## 3. Results and Discussion

### 3.1. Sorption Isotherm Experiments

**Figure 1:**
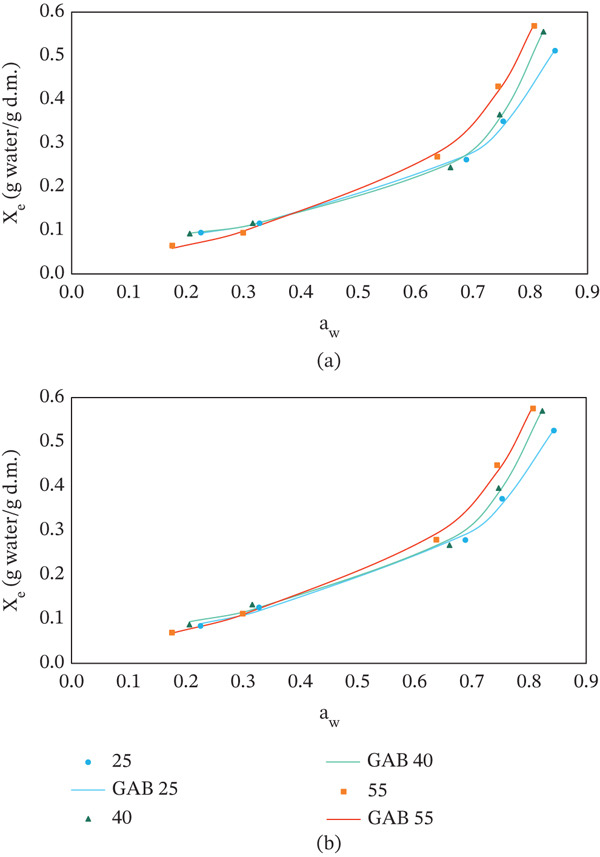
Experimental data of equilibrium moisture content for *C. chamissoi* and the predictive model with the GAB model at 25°C (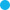), 40°C (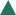), and 55°C (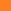). (a) Tetrasporophyte and (b) carposporophyte.

Figure 1 illustrates the experimental sorption data and the desorption curves. According to the van der Waals classification of isotherms [34], the tendency described by the isotherm graphics could be classified as Type III. Seaweeds such as *M. pyrifera* [17] and *B. bifurcata* [30] have Isotherm Type II, while *Gracilaria* sp. [25] and *F. vesiculosus* [29] have Isotherm Type III. Both types are associated with high sugar, polysaccharide, and protein products, because at low water activity (*a*_*w*_), water molecules strongly associate with biopolymers [35].

Figure 1 presents the experimental EMC and GAB model fits for *C. chamissoi* in both reproductive phases. In Figure 1A,B, the EMC increases in relation to water activity (*a*_*w*_), displaying a sigmoidal isotherm characteristic of hydrophilic macroalgal matrices.

In the tetrasporophyte phase (Figure 1A), the increase in EMC is moderate at low and intermediate *a*_*w*_ values (≤ 0.6), whereas a more pronounced increase occurs from *a*_*w*_ ≈ 0.65–0.70 onward. This change in slope suggests greater availability of sorption sites associated with multilayer water or macromolecular dissolution phenomena. A clear temperature effect is also observed: at a given *a*_*w*_, EMC tends to be higher at 55°C, followed by 40°C and 25°C, particularly in the high *a*_*w*_ range.

In the carposporophyte phase (Figure 1B), a similar trend is observed, with a gradual increase in EMC at low *a*_*w*_ and a marked acceleration at higher *a*_*w*_ values. However, compared to the tetrasporophyte, the carposporophyte exhibits slightly higher EMC values in the high *a*_*w*_ range, which may be related to structural or compositional differences between the two reproductive phases. The effect of temperature is again evident, with higher EMC at 55°C.

In both cases, the GAB model adequately describes the experimental data, showing good agreement between experimental values and predicted curves across the entire range of *a*_*w*_ and temperatures studied. Overall, the figure confirms that the GAB model is suitable for describing the variation in EMC of *C. chamissoi* in both reproductive phases and highlights the combined influence of *a*_*w*_ and temperature on sorption behavior.

A crossover point was also observed in both stages: approximately at 0.4 *a*_*w*_ for the 55°C isotherm compared to the other two temperatures, and another crossover point between 25°C and 40°C was identified within the *a*_*w*_ range of 0.6–0.7. After this crossover point, the influence of temperature is inverted, leading to higher EMC at elevated temperatures in both scenarios.

This shift in the initial trend may be attributed to the high polysaccharide and protein content of the seaweed and their solubility [29]. Tabachi and García [3] reported a carbohydrate content of 41.34% (d.m.) in *C. chamissoi*, while Vásquez et al. [4] described the following proximate composition: 49.26*%* ± 0.36*%* (d.m.) carbohydrates, 17.60*%* ± 1.0*%* (d.m.) protein, 1.44% (d.m.) crude fiber, 2.21% (d.m.) lipids, and 21.78% (d.m.) ash, highlighting the high sugar content of this species. Neither study specified the reproductive stage of the analyzed *C. chamissoi* samples. After the crossing point, the solubility of carrageenan and proteins may be influenced by relative humidity, particularly given the high proportion of these macromolecules reported in the literature. However, because no direct compositional measurements were performed in the present study, future research should include detailed compositional and structural analyses of carrageenan and proteins to confirm their role in moisture sorption and dehydration behavior.

It can be hypothesized that the polysaccharides present in *C. chamissoi* (which may occur not only as free carrageenan types but also as hybrid and more complex structures such as CCC, a mixture of *κ*‐, *ι*‐, and *μ*‐carrageenan) [6] could contribute to a more complex dehydration behavior after the crossing point [24, 25, 29, 30].

### 3.2. Modeling of Desorption Isotherms

The parameters obtained from the GAB, BET, Halsey, and Oswin models are shown in Table 3. The GAB equation was the best fit for the experimental desorption phenomena for both reproductive stages, with the highest *R* ^2^ values (0.9988 for tetrasporophyte at 55°C and 0.9985 for carposporophyte at 40°C) and lowest RMSE values (0.0058 for tetrasporophyte at 55°C and 0.0075 for carposporophyte at 40°C). The remaining models also presented a good fit, especially BET at 55°C for the tetrasporophyte, with a difference of 0.0000003 in *R* ^2^ and 0.0000023 in RMSE. Hence, the GAB sorption model best describes the equilibrium moisture data for all three temperatures as shown in Figure 1.

**Table 3:** Parameters and constants of the sorption models at 25°C, 40°C, and 55°C.

Model	Constant	Tetrasporophyte			Carposporophyte		
		25°C	40°C	55°C	25°C	40°C	55°C
GAB	*C*	12.9790	6.7442	40.0323	13.1807	3.2383	3.9710
	*K*	0.9753	0.9504	1.0410	1.0145	1.0008	0.9910
	*M*_*o*_	0.0926	0.1085	0.0800	0.0957	0.1181	0.1236
	*R* ^2^	0.9982	0.9971	0.9988	0.9971	0.9985	0.9975
	RMSE	0.0066	0.0087	0.0058	0.0095	0.0075	0.0097

BET	*C*	39.0629	23.8582	8.1690	9.2952	3.2050	4.4206
	*M*_*o*_	0.0823	0.0862	0.0978	0.1024	0.1186	0.1186
	*R* ^2^	0.9958	0.9894	0.9925	0.9965	0.9985	0.9974
	RMSE	0.0102	0.0171	0.0149	0.0105	0.0075	0.0099

Halsey	*A*	0.0735	0.0763	0.1025	0.1031	0.1247	0.1228
	*B*	1.2554	1.2688	1.0564	1.1137	0.9834	1.0375
	*R* ^2^	0.9980	0.9960	0.9929	0.9953	0.9984	0.9971
	RMSE	0.0070	0.0103	0.0152	0.0123	0.0078	0.0105

Oswin	*A*	0.1716	0.1804	0.1676	0.1863	0.1801	0.1956
	*B*	0.6385	0.6314	0.7549	0.7098	0.8019	0.7535
	*R* ^2^	0.9928	0.9957	0.9812	0.9875	0.9979	0.9965
	RMSE	0.0137	0.0107	0.0254	0.0208	0.0093	0.0117

The GAB equation has also shown to be the best to describe the isotherms of *G. sesquipedale* [19], *M. pyrifera* [17], and *U. ohnoi* [24].

The GAB as well as the BET equation has a value of special importance, the *M*_*o*_ or monolayer parameter. This value represents the water molecules at the primary layer, meaning that at that level of water content, the loss of quality due to physical, chemical, or microbial factors is negligible [36]. For the tetrasporophyte stage, our values ranged from 8.00% to 11.81% dry matter, being from 9.57% to 12.36% dry matter for the carposporophyte stage. These values are comparable to the average *M*_*o*_ values obtained for other seaweeds: 8.97% for *G*. *sesquipedale*, 18.7% for *M. pyrifera*, and 7.47% for *U. ohnoi*. Determination of this value allows the calculation of a drying target for *C. chamissoi* and thereby avoids overuse of energy for drying.

The *K* and *C* parameters are related to the sorption at a multilayer and monolayer level, respectively [37]. Quirijns et al. [38] defined the *K* parameter as a correction factor, providing better representation. The *C* parameter represents how strongly the water is bound in the monolayer. At 55°C, the value decreases, indicating a lower strength of the water binding, probably due to the excitation of the molecules, leading to a greater physical separation between the molecules.

### 3.3. Experiment of Drying Kinetics

The initial moisture content (*X*_0_) was 9.25 g water/g d.m. for tetrasporophytes and 8.24 g water/g d.m. for carposporophytes. The GAB equation was used to calculate the EMC for all the temperatures in both phases. For tetrasporophytes, the EMC decreased from 0.11 g water/g d.m. at 40°C to 0.09 g water/g d.m. at both 50°C and 60°C and remained constant at 0.09 g water/g d.m. at both 60°C and 70°C. In carposporophytes, the EMC values were 0.09, 0.07, 0.04, and 0.04 g water/g d.m. at 40°C, 50°C, 60°C, and 70°C, respectively. All the drying processes achieved a value < 18%, thereby providing commercial stability for the product [25].

**Figure 2:**
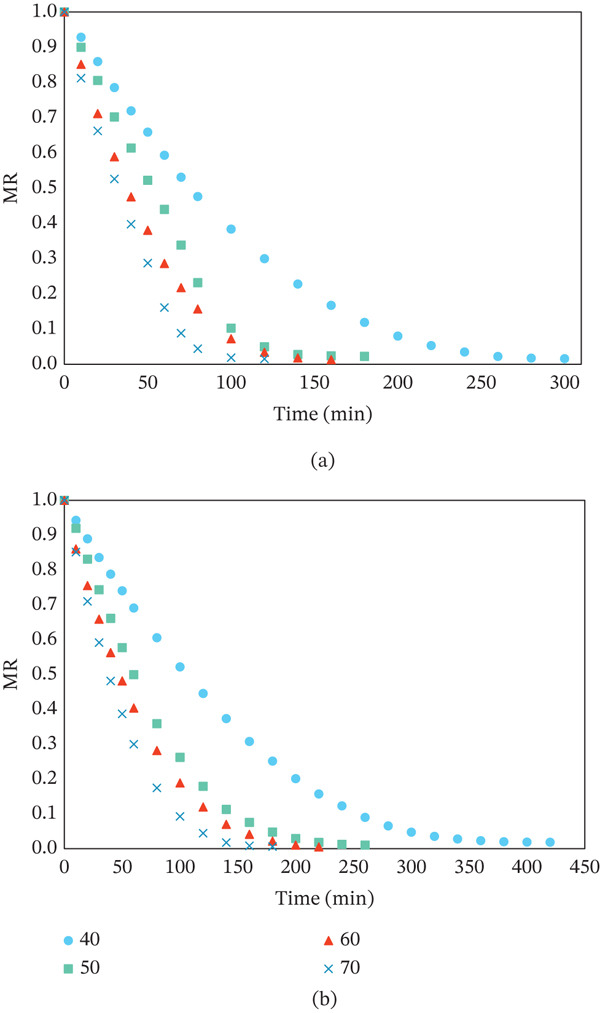
Drying curves for the red algae *Chondracanthus chamissoi* at different temperatures. (a) Tetrasporophyte. (b) Carposporophyte.

Figure 2 shows the drying curves of the seaweed under controlled parameters. We first see that there is only a falling rate period, and the constant drying rate is not present. We also observe in both cases that drying time shortens when temperature increases, but drying time was different for tetrasporophytes and carposporophytes at the same temperature.

The drying time observed at 70°C was 120 min for the tetrasporophytes and 180 min for the carposporophytes. The same pattern occurred in the tetrasporophytes and carposporophytes, respectively, at the following temperatures: 160 and 220 min at 60°C, 180 and 260 min at 50°C, and 300 and 420 min at 40°C. Given that the only difference between the samples is the presence of the cystocarps on the pinnules of the carposporophyte, the longer drying time is likely related to this structure, considering that all the samples started with 10 ± 0.5 g.

Figures 1 and 2 show an exponential tendency, recommending the use of the proposed models (Newton, Page, Modified Page, and Henderson–Pabis). Similar plots have previously been obtained for other seaweeds including *G. chilensis* [18, 25], *M. pyrifera* [17], and *H. elongata* [31].

### 3.4. Modeling for Drying Kinetic Curves

Table 4 shows the means and standard deviations for the kinetic parameters of the models tested. The *k*_*i*_ (*i* = 1, 2, 3) values increased with the drying temperature. Various authors have described the direct influence of temperature on *k*_*i*_ in other macroalgae, such as G. *chilensis* [20], *M. pyrifera* [17], *Irish brown* [31], and *B. bifurcata* [30].

**Table 4:** Values of kinetic parameters for each model by drying curve.

**Tetrasporophyte**			
**T (°C)**	**Newton**	**Modified Page**	**Henderson–Pabis**
	**k**_1_ × 10^−2^	**k**_2_ × 10^−2^	**k**_3_ × 10^−2^

40	1.009 ± 0.0008 a	1.003 ± 0.0008 a	1.079 ± 0.0008 a
50	1.574 ± 0.0007 b	1.572 ± 0.0006 b	1.714 ± 0.0007 b
60	2.058 ± 0.0015 c	2.025 ± 0.0014 c	2.159 ± 0.0015 c
70	2.604 ± 0.0020 d	2.516 ± 0.0019 d	2.737 ± 0.0020 d

**Carposporophyte**			
**T (°C)**	**Newton**	**Modified Page**	**Henderson–Pabis**
	**k**_1_ × 10^−2^	**k**_2_ × 10^−2^	**k**_3_ × 10^−2^

40	0.754 ± 0.0002 a	0.738 ± 0.0002 a	0.802 ± 0.0003 a
50	1.307 ± 0.0024 b	1.298 ± 0.0024 b	1.411 ± 0.0025 b
60	1.592 ± 0.0011 b	1.580 ± 0.0011 b	1.650 ± 0.0011 b
70	2.021 ± 0.0030 c	2.006 ± 0.0030 c	2.115 ± 0.0027 c

*Note:* Data are expressed as average ± standard deviation in triplicate. Values in the same column having the same letter for each parameter do not have statistically significant differences at a confidence level of 95%.

Therefore, when the ANOVA test was run for the *k*_*i*_ parameter, a *p* < 0.05 was recorded for each case, verifying that this parameter is dependent on the temperature. The posterior MRT for *k*_1_, *k*_2_, and *k*_3_ allowed multiple comparisons to be made between the means at each temperature and determined if they differ. The test shows that the *k* of each model was statistically different for each temperature. For tetrasporophytes, all *k*_*i*_ formed four nonhomogeneous groups, and for carposporophytes, three groups (Table 4).

**Figure 3:**
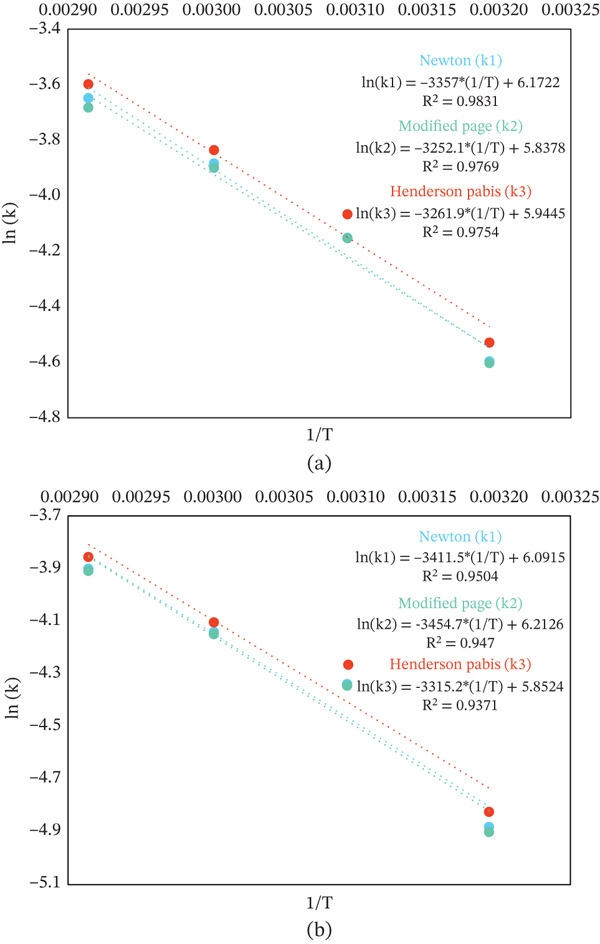
Graphic representation of the influence of absolute temperature (Kelvin) for different models and kinetic parameters. (a) Tetrasporophyte. (b) Carposporophyte.

On observing that the means of each *k*_*i*_ were different, another test was conducted to confirm temperature dependence, using an Arrhenius‐type equation (Figure 3). The values of *r* ^2^ for tetrasporophyte and carposporophyte were 0.97 and 0.93, respectively. This equation can also help to establish the activation energy (*E*_*a*_) for each model [33]. *E*_*a*_ represents the amount of energy necessary to overcome for a reaction to occur [39]. The greater the value, the more energy the reactant requires to start its motion.

The *E*_*a*_ obtained for *k*_1_, *k*_2_, and *k*_3_ were 27.91, 27.04, and 27.12 kJ/mol for the tetraspororic group and 28.36, 28.72, and 27.56 kJ/mol for *k*_1_, *k*_2_, and *k*_3_ of the carposporophyte group. The *E*_*a*_ values of *C. chamissoi* were lower than those of *G. chilensis*, *H. elongata*, and *U. ohnoi* (Table 5), and thus, the dehydration process is easier to achieve in *C. chamissoi* than in the other seaweeds.

**Table 5:** Values of activation energy for different macroalgae.

Seaweed	*E*_*a*_ (kJ/mol)	Reference
*C. chamissoi* (tetrasporophyte)	27.04–27.91	The present study
*C. chamissoi* (carposporophyte)	27.56–28.72	The present study
*Gracilaria chilensis*	33.49–33.85	[27]
*Gracilaria* sp.	12.27–38.60	[39]
*Macrocystis pyrifera*	25.89–78.74	[17]
*Gracilaria chilensis*	28.35	[18]
*Himanthalia elongata*	41.8–46.7	[31]
*Ulva ohnoi*	41.3	[38]

Table 6 shows the empirical parameters of mathematical models. With the empirical addition of dimensionless *n* and *a* constant to the drying equations, the error is minimized [40]. The *n* values obtained in the Modified Page equation ranged from 1.25 to 1.49 in the tetrasporophyte group and from 1.17 to 1.33 in the carposporophyte group. On comparison with other seaweeds, the *n* values of *Gracilaria* ranged from 0.96 to 1.02 [25], while the mean value for *M. pyrifera* was 1.25 [17] and that of Nori made of *Ulva lactuca* mixed with *Gracilaria* sp. was 1.56 [41], like the values recorded for *C. chamissoi* in the present study. The “*a*” parameter ranged from 1.05 to 1.08 for tetrasporophyte and 1.03 to 1.07 for carposporophyte, like values found in *Gracilaria* (1.03–1.06), *M. pyrifera* (1.24), and *H. elongata* (0.98–1.06) [17, 27, 31].

**Table 6:** Values of empirical parameters for each model by drying curve.

**Tetrasporophyte**		
**T (°C)**	**Modified Page**	**Henderson–Pabis**
	**n**	**a**

40	1.256 ± 0.0504 a	1.062 ± 0.0106 ab
50	1.491 ± 0.0687 b	1.082 ± 0.0065 a
60	1.253 ± 0.0369 a	1.048 ± 0.0054 b
70	1.371 ± 0.1474 ab	1.054 ± 0.0209 b

**Carposporophyte**		
**T (°C)**	**Modified Page**	**Henderson–Pabis**
	**n**	**a**

40	1.253 ± 0.0399 ab	1.058 ± 0.0081 ab
50	1.325 ± 0.0296 b	1.073 ± 0.0082 a
60	1.166 ± 0.0447 a	1.033 ± 0.0109 c
70	1.236 ± 0.1055 a	1.045 ± 0.0197 cb

*Note:* Data are expressed as average ± standard deviation in triplicate. Values in the same column having the same letter for each parameter do not have statistically significant differences at a confidence level of 95%.

The same ANOVA was performed for the empirical parameters at a significance of 5%. For tetrasporophyte, *n* and *a* had a *p* value < 0.05, indicating that temperature had a significant effect on the empirical parameters. For carposporophyte, the *p* value of *n* was > 0.05, and that of *a* was < 0.05, indicating that temperature only influences *a*.

Nonetheless, when the MRT test was carried out for tetrasporophytes, two groups were identified for each parameter and three groups for *a* parameter in carposporophytes. The grouping in this case contrasts with the nonhomogeneous groups formed with the kinetic parameters. The groups are fewer and do not follow a temperature pattern. Other authors have found that the empirical parameters of the Modified Page (*n*) and Henderson–Pabis (*a*) models show no statistical significance in relation to temperature and hypothesize that they are related to other factors such as type of tissue, drying air flow, and initial moisture [42] and are therefore considered to be constants for the type of product used [27].

Finally, Table 7 shows that all three models provided a good fit to the experimental data for both reproductive phases across the entire temperature range, with *R* ^2^ values higher than 0.97 in most cases. However, clear differences in the relative performance of the models were observed. The Modified Page model showed the best overall fit for both the tetrasporophyte and the carposporophyte. This model consistently exhibited the highest *R* ^2^ values (approximately 0.994–0.999) and the lowest SSE and RMSE values at all evaluated temperatures, indicating excellent capability to describe the drying kinetics. At 40°C and 60°C, the lowest error values were obtained for both phases, confirming the robustness of the model under different temperature conditions. Overall, these results indicate that the Modified Page model is the most suitable for describing the drying behavior of both reproductive phases over the studied temperature range, while the Newton and Henderson–Pabis models can be considered acceptable but less accurate alternatives.

**Table 7:** Statistical tests of the Newton, Modified Page, and Henderson–Pabis equations for each drying temperature.

Model	*T* (°C)	Tetrasporophyte			Carposporophyte		
		SSE	RMSE	*r* ^2^	SSE	RMSE	*r* ^2^
Newton	40	0.0356	0.0405	0.9942	0.0422	0.0418	0.9935
	50	0.0639	0.0681	0.9775	0.0392	0.0484	0.9921
	60	0.0185	0.0375	0.9932	0.0131	0.0282	0.9965
	70	0.0351	0.0555	0.9815	0.0185	0.0354	0.9943

Modified Page	40	0.0031	0.0116	0.9988	0.0057	0.0156	0.9982
	50	0.0087	0.0250	0.9948	0.0020	0.0109	0.9991
	60	0.0020	0.0122	0.9986	0.0028	0.0131	0.9986
	70	0.0078	0.0267	0.9937	0.0020	0.0115	0.9988

Henderson–Pabis	40	0.0245	0.0336	0.9916	0.0304	0.0362	0.9908
	50	0.0498	0.0601	0.9718	0.0266	0.0399	0.9889
	60	0.0145	0.0331	0.9911	0.0108	0.0257	0.9953
	70	0.0301	0.0515	0.9781	0.0144	0.0313	0.9923

## 4. Conclusions

The EMC of *C. chamissoi* was found using the static gravimetric method. We concluded that the isotherms recorded under 25°C, 40°C, and 55°C belong to the Type III isotherm according to the BET classification for both reproductive stages. Among the four equations, the GAB model was the most accurate for determining the behavior of the isotherm with the lowest SSE, RMSE, and highest *r* ^2^. For the drying kinetics, four temperatures were evaluated, 40°C, 50°C, 60°C, and 70°C, showing a clear exponential tendency with no constant rate period. The Modified Page equation had the best fit with the experimental MR in both the tetrasporophyte and carposporophyte phases according to the same statistical test and therefore represents the best tool for the prediction of drying time. The kinetic parameters *k*_*i*_ (*i* = 1, 2, 3) were temperature‐dependent according to the Arrhenius‐type equation, and the *E*_*a*_ values obtained were 27.93, 27.01, and 27.16 kJ/mol for tetrasporophytes and 28.07, 28.47, and 20.97 kJ/mol for *k*_1_, *k*_2_, and *k*_3_ for carposporophytes, respectively.

## Funding

This work was supported by the PNIPA‐PES‐SIADE‐PP‐000021 Project “Agregación del valor Comercial para el Recurso Algas rojas (Chondracanthus Chamissoi y Porphyra sp) mediante el Desarrollo de Productos para el Consumo Humano” and the Vicerrectorado de Investigación of the Universidad Científica del Sur (No. REGISTRO: 840‐2020‐PREB5) where the work was executed.

## Conflicts of Interest

The authors declare no conflicts of interest.

## Data Availability

The data that support the results of the present study are available upon request to the authors.
